# Trends and correlates of intimate partner violence experienced by ever-married women of India: results from National Family Health Survey round III and IV

**DOI:** 10.1186/s12889-021-12028-5

**Published:** 2021-11-05

**Authors:** Priyanka Garg, Milan Das, Lajya Devi Goyal, Madhur Verma

**Affiliations:** 1 Department of Obstetrics and Gynaecology, All India Institute of Medical Sciences, Bathinda, Punjab 151001 India; 2 International Institute for Population Sciences, Mumbai, India; 3 Department of Community and Family Medicine, All India Institute of Medical Sciences, Bathinda, Punjab 151001 India

**Keywords:** Violence, Intimate partner violence, Mental health, Women health, National family health survey

## Abstract

**Background:**

The study aims to estimate the prevalence of Intimate partner violence (IPV) in India, and changes observed over a decade as per the nationally representative datasets from National Family Health Surveys (NFHS) Round 3 and 4. We also highlight various socio-demographic characteristics associated with different types of IPV in India. The NFHS round 3 and 4 interviewed 124,385, and 699,686 women respondents aged 15–49 years using a multi-stage sampling method across 29 states and 2 union territories in India. For IPV, we only included ever-married women (64,607, and 62,716) from the two rounds. Primary outcomes of the study was prevalence of the  ever-experience of different types of IPV: physical, emotional, and sexual violence by ever-married women aged 15 to 49 years. The secondary outcome included predictors of different forms of IPV, and changes in the prevalence of different types of IPV compared to the previous round of the NFHS survey.

**Results:**

As per NFHS-4, weighted prevalence of physical, sexual, emotional, or any kind of IPV ever-experienced by women were 29.2%, 6.7%, 13.2%, and 32.8%. These subtypes of IPV depicted a relative change of − 14.9%, − 30.2%, − 11.0%, − 15.7% compared to round 3. Significant state-wise variations were observed in the prevalence. Multivariate  binary logistic regression analysis highlighted women's and partner’s education, socio-economic status, women empowerment, urban-rural residence, partner’s controlling behaviours as major significant predictors of IPV.

**Conclusions:**

Our study findings suggest high prevalence of IPV with state-wise variations in the prevalence. Similar factors were responsible for different forms of IPV. Therefore, based on existing evidences, it is recommended to offer adequate screening and counselling services for the couples, especially in health-care settings so that they speak up against IPV, and are offered timely help to prevent long-term physical and mental health consequences.

**Supplementary Information:**

The online version contains supplementary material available at 10.1186/s12889-021-12028-5.

## Strengths and limitations of the study

- One of the very first comprehensive assessment of the different types of IPV using data from the third and fourth rounds of the National Family Health Survey India.
- Predictors of IPV were estimated through a weighted analysis, that helped in highlighting certain feasible actionable points
- Large and nationally representative data on violence were analysed from India.
- Appropriate sampling during the survey makes the results generalisable, and recommendations can be adopted by other Lower Middle Income countries.
- The use of only the predetermined variables to predict IPV is the key limitation to this analysis as there are many other variables apart from those included in the NFHS that affect IPV.
- Lastly, self-reporting may under-estimate the overall prevalence of the IPV, owing to the fear, economic dependence, humiliation, and the feeling of severe confinement by the women.

## Introduction

Spousal or Intimate partner violence (IPV) is one of the most common forms of domestic violence (DV), and refers to any physically, psychologically, sexually, or economically harmful behavior in an intimate relationship [1]. The three levels of IPV are Level I abuse (pushing, shoving, grabbing, throwing objects to intimidate, or causing damage to property and pets), Level II abuse (kicking, biting, and slapping), and Level III abuse (use of a weapon, choking, or attempt to strangulate) [2]. IPV takes place across different age groups, genders, sexual orientations, economic, or cultural status. The World Health Organization (WHO) recently estimated that almost one-third of women who have been in a relationship have experienced IPV [3]. Other studies have depicted the prevalence of IPV in the range of 13 - 61% in women (15–49 years old) [3]. As per the National Family Health Survey (NFHS) round 4, the prevalence of IPV ranges between 3 - 43% in different states of India. Marital violence acceptability is amongst the highest in the world (52% women, and 42% men) [4]. The magnitude of IPV is underestimated as many studies indicate the difficulty of obtaining clear figures about prevalence of IPV in general population [5, 6]. This is because of the under reporting which can be attributed to the fear of reprisal by the perpetrator, economic dependence on the spouse, a hope that IPV will stop, humiliation, loss of social prestige, and the feeling of severe confinement. However, there are anecdotal evidences which suggest that approximately nine out of ten of victims of IPV don’t disclose such mis-happenings and suffer all alone.

Ecological model has been preferred by many scientists around the world to understand IPV, according to which violence is an outcome of interaction between multiple causal agents operating at individual, relationship, community and societal levels [3]. There are various culturally specific norms that exist at these levels. These norms offer social standards of appropriate and inappropriate behavior that may favor or discourage IPV [7]. For instance, India had a deep-rooted patriarchal society, with preference to male child. It has been observed that Indian states with anisometric sex-ratios of first births favouring males, women with first born sons are less likely to experience IPV than those with first born daughters and, among those who have experienced IPV once, are more likely to experience it again [8]. Then, there are societal issues that act as perpetrators of IPV like dowry, inequities in education, and decision making powers. Spousal factors like alcohol, and other substance abuse, unemployment, challenges to masculinity norms are significant factors. At individual levels, IPV is more pronounced among less educated and poor women. High level of IPV and its acceptability in society corroborates with other factors that point towards gender discrimination and other social inequalities [9]. All these factors depicts the link between sex-discrimination and IPV at the household level, which is bolstered in an environment where females are regularly downplayed [10, 11].

There is a substantial evidence suggesting that IPV may act an causal agent to a plethora of acute and chronic physical, mental, and sexual health problems [12]. Abused women commonly suffer from chronic gynecological problems, including chronic pelvic pain, sexually-transmitted diseases and vaginal bleeding, and present very frequently to healthcare services and require a wide range of medical services [13]. Other conditions affecting abused women include chronic pain such as back pain and headaches, neurological symptoms such as fainting and seizures, and gastrointestinal disorders such as irritable bowel disease [14, 15]. IPV is also a significant risk to pregnant women and their unborn children. The WHO recently reported that abused women have two times higher chances of having an abortion, miscarriage, premature birth, fetal injury, and low birth weight baby [16–19]. Apart from physical injuries, abused women have a lot of mental health problems like depression, anxiety, post-traumatic stress disorder and substance abuse [20–22]. Such women also suffer from low self-esteem and hopelessness [23]. These problems impact upon women’s ability to parent their children [24]. In addition, there are also wider economic societal implications of IPV that needs to be considered.

Around the world, considerable attention is being given to IPV. For instance, European Union Agency for Fundamental Rights affirmed in 2015 that IPV can be perceived as an infringement of human rights and dignity [25] . On similar lines, the United States Department of Justice stated that IPV has a considerable impact on victim, as well as family members, and other acquaintance of both the abuser and the victim [26]. In this sense, children who witness violence while growing up can be severely emotionally damaged. In India, IPV has been recognized since 1983 as a criminal offense under Section 498-A of the Indian Penal Code, and is comprehensively defined in the Protection of Women from Domestic Violence Act (PWDVA) 2005, which came into effect in 2006 [27]. Even after the enactment of the Act, over the last decade, the rate of decline in IPV prevalence has remained abysmally low in India.

Management of IPV demands a need for multi-pronged collaboration between different stakeholders at various levels of the ecological framework. Though, individual-level interventions are comparatively easy to assess, evaluation of multi-component programmes and institutional-wide reforms is more challenging, and are also the most under-explored [12]. The existing literature describes all kind of violence comprehensively, and doesn’t attempt to explain the differences in the socio-demographic variations observed in the various forms of violence like physical, sexual and emotional type. Each type of IPV may have  its own correlates, and management strategies. Hence, they need to be studied separately. For instance, similar counselling sessions cannot be given to women experiencing sexual violence by alcoholic husband and emotional violence instigated by the non-earning husband. Health care providers can play a pertinent role in identifying women who are experiencing IPV and halting the agonizing cycle of abuse through screening, timely support, and offering suitable prevention and referral options [28]. They are often the first professionals to offer care to women facing IPV. Therefore, this study attempts to explain the experience of IPV in India, and changes observed in the prevalence of various forms of IPV after the enactment of the PWDVA 2005, through the use of nationally representative datasets from NFHS round 3 and 4. We will also attempt to highlight various socio-demographic characteristics associated with physical, sexual and emotional type of IPV in India. The results of such analysis using a national survey holds merits compared to a sub-national estimates, to give a comprehensive picture about the IPV, and deduce meaningful interpretations for advocacy, and policy making.

## Methodology

### Study design

A repeated, independent, cross-sectional ecological study design was used in the present study.

#### Data source

The present study uses data from the third and fourth rounds of the NFHS conducted in 2005–06 and 2015–16. The NFHS is India’s version of the Demographic and Health Survey (DHS). In NFHS-3, all 28 states and New Delhi were covered, while NFHS included all 29 states and all union territories using a multistage stratified cluster sampling procedure for data collection. The NFHS-3 and 4 included women and men aged between 15–49 and 15-54 years in the primary sample. The design of the study, sampling strategy, and other details of the NFHS-3 and 4 can be found in the NFHS report (IIPS & ICF International, 2007,2017). The surveys collected information on child and maternal health, family welfare and domestic violence including  IPV.

The NFHS follows both Indian and international guidelines, e.g. WHO ethical guidelines for research on domestic violence against women, 2001, for the ethical collection of data on violence. NFHS-4 sample size was approximately 699,686 women, up from about 124,385 women of NFHS-3. Domestic violence related questions were included in the state module, where about 68% in NFHS-3 and 15% in NFHS-4 of the total sample was selected for the interview. It should be noted that to assess DV, a total of 84,703 women (never-married 14,219, ever-married 64,607, others 5877) were interviewed during NFHS-3 survey, while a total of 79,729 women (never-married 13,716, ever-married 62,716, others 3297) were interviewed during NFHS-4 survey.

#### Study participants

From each household, only one woman was  invited to complete the DV module, and sample weights, specific to the estimation of DV, were calculated to adjust for the selection and ensure that the DV subsample is nationally representative. Of all the women who were invited for the DV module, we only included 64,607 and 62,716 ever married women from the round 3 and 4 in our analysis.

#### Study variables

##### Dependent variables

The main dependent variables for this analysis are the ever-experience of different types of IPV: physical, emotional, and sexual violence by a partner of ever-married women aged 15 to 49 years. In both the NFHS-3 and-4, DV is defined to include violence by spouses as well as by other household members [29, 30]. However, it is well documented that IPV is one of the most common forms of violence experienced by married women [3].

The set of questions in NFHS survey attempts to capture detailed information on physical, sexual and emotional IPV. Information is obtained from ever-married women on violence by husbands and by others, and from never married women on violence by anyone, including boyfriends. In NFHS-3 & 4 surveys, spousal physical, sexual and emotional violence for ever-married women is measured using the following module of questions.

##### Physical violence

Physical IPVs was defined as any type of physical violence experienced by a woman at the hands of husband/partner, which includes: (a) ever having been slapped; (b) ever having had arm twisted or hair pulled; (c) ever having been pushed, shaken or had something thrown at them; (d) ever having been punched with fist or hit by something harmful; € ever having been kicked or dragged; (f) ever having been strangled or brunt; (g) ever having been threatened with knife/gun or other weapon.

##### Sexual violence

The Sexual IPVs was captured by three questions: a) ever having been physically forced you to have sexual intercourse with him even when you did not want to; b) ever having been physically forced you to perform any other sexual acts you did not want to c) ever having been forced you with threats or in any other way to perform sexual acts you did not want to.

##### Emotional violence

(a) ever having been said or done something to humiliate you in front of others b) ever having been threatened to hurt or harm you or someone close to you c) ever having been insulted you or make you feel bad about yourself.

The expected responses to all the above questions were coded as either ‘never’, ‘often’, ‘sometimes’, ‘yes but not in the last 12 months’. Of these, all response except ‘never’ to the questions related to IPVs implied experience of physical, sexual and emotional violence respectively. For the ease of analysis, all responses except ‘never’ were coded as Yes = 1, while never was coded as No = 0.

#### Independent variables

The independent variables were categorized as per the women’s individual, partner, family level factors. The selection of these variables were based upon extensive literature review. All those variables that have been highlighted in previous studies from India and abroad, and were available in the DHS datasets were included in our study [11, 27, 31–34]. Individual level factors included her age, education status, age at first marriage, parity, and economic empowerment status; and partner level factors included his education status, controlling behavior, and history of substance abuse like alcohol. Family factors included duration of cohabitation in years, number of co wives, and history of witnessing parental violence, while the household and community level factors included the wealth status, religion, place of residence, and region of the country.

These variables were categorized as age (15–19, 20–24, 25–34, 35+), educational attainment (no education, primary, secondary and higher), wealth quintile status (poorest, poorer, middle, richer, richest), religion (Hindu, non-Hindu), place of residence (urban, rural), region of India classified as North, Central, East, Northeast, West, and South. The North region includes Jammu & Kashmir, Himachal Pradesh, Punjab, Chandigarh, Uttarakhand, Haryana and Delhi; the Central region includes, Rajasthan, Uttar Pradesh, Chhattisgarh and Madhya Pradesh; the East region includes West Bengal, Jharkhand, Odisha and Bihar; the North-east region includes Sikkim, Arunachal Pradesh, Nagaland, Manipur, Mizoram, Tripura, Meghalaya and Assam; the West region includes Gujarat, Maharashtra, Goa; and finally, Andhra Pradesh, Telangana, Karnataka, Kerala, Tamil Nadu and Puducherry belong to the South region. Parity was categorized as 0 = None, 1 = 1–2 parity,2 = 3+ parities. Economic empowerment was considered if women had ownership of property (house and land) or was gaining earnings from her work. It was obtained by merging women responses to questions: does a respondent: a) own a house? b) own land [either alone or jointly with a partner for both questions a) and b)] and c) type of earning from her work. The analysis dichotomized question c into paid (cash only, cash and in kind, and in kind only) and not paid. Any one of the three questions a, b, or c indicated a ‘yes’ that a woman is considered empowered and ‘no’ meant non empowerment. Responses to these questions were recorded into two categories (0 = Not empowered, 1 = Empowered).

The variable ‘Age at marriage’ was categorized as 0 = less than or equal to 18 years, 1 = more than 18 years. ‘Witnessing parental violence’ was measured by a question that asked- ‘whether the respondent’s father had ever beat her mother’, which was recorded dichotomously (0 = NO, 1 = Yes). ‘Duration of cohabitation in years’ was categories as 0 = 0–4, 1 = 5–9, 2 = 10–14,3 = 15–19 and 4 = 20 + years. ‘Number of co-wives’ were categories as 0 = None, 1 = One and more.

To measures the partner’s controlling behavior, respondents were asked- “Does your partner ever or did; a) Prohibit you to meet female friends? b) Limit you contact your family? c) Insist on knowing where you are at all times? d) Is jealous if you talk with other men? And e) Frequently accuses you of being unfaithful?” These questions were merged into one variable the “partner’s controlling behaviors”. Any positive response (yes) to any of the above questions implies the existence of such behavior and no to all the questions implied nonexistence of such behaviors. The partner’s alcohol consumption was measured by responses to the questions, “Does your partner drink alcohol?” and it had a binary outcome (0 = No, 1 = Yes). Frequently of a partner being drunk was follow-up question to those respondents whose partners indicated that the partner drank alcohol.

#### Patient and public involvement

No patients were involved in the study.

#### Data analysis

Due permissions were sought from the Demographic and Health Survey program for data access and analysis after submitting the protocol and study objectives [35]. NFHS-3 and 4 datasets were imported into Stata version 14 for analysis. We calculated the weighted prevalence of each type of IPVs ever experience by the female respondents by doing univariate analysis separately for NFHS rounds 3 and 4 to get nationally representative estimates. Logistic regressions were used to estimate the unadjusted (OR) and adjusted odds ratio (aOR) with 95% confidence interval (CI) to depict the association of ever experience of different types of IPVs by the respondent with the independent variables. We used the domestic violence weighting variable (d005) included in the NFHS data and the Stata survey (svy) command to weight the data during the analysis in order to account for the complex survey design. We also explored the relative change in the different types of IPVs between two rounds of NFHS surveys in India.

#### Ethics approval and consent to participate

This is a secondary analysis of a nationally representative survey dataset NFHS-4 (2015–16) which is in public domain. The Institute Ethics Committee of All India Institute of Medical Sciences, Bathinda waived off the need for ethical clearance for this study wide letter no. IEC/AIIMS/BTI/032.

## Results

Table 1 depicts the socio-demographic characteristics of the 64,607 and 62,716 ever married females who consented to respond to the domestic violence module of NFHS-3 and 4. The sample in both the rounds was comparable in terms of age distribution, region of country they belong to, wealth status, religion, parity, and duration of cohabitation after marriage. However, a higher number of respondents were educated in round 4 (54.2%), while non-educated group was prevalent as per the 3rd round (47.2%). Most of the respondents from round 4 were economically empowered (55.8%). Nearly four-fifth of the them had witnessed parental violence, and more than half were experiencing partners controlling behavior. Age of marriage of the respondents had shifted primarily from < 18 Years in round 3 to > 18 years in round 4.

**Table 1:** Characteristics of the women who were interviewed for the domestic violence module during the National Family health Surveys Round 3 and 4, India

Background Characteristics	NFHS-3	NFHS-4
	Unweighted Frequency (weighted percentage)	Unweighted Frequency (weighted percentage)
**Total**	64,607 (100)	62,716 (100)
**Age of the respondent**		
15–19	4702 (7.3)	2253 (3.6)
20–24	11,674 (18.1)	9701 (15.5)
25–34	12,923 (20)	12,612 (20.1)
35+	35,307 (54.7)	38,150 (60.8)
**Respondents education status**		
No education	30,485 (47.2)	19,892 (31.7)
Primary	9823 (15.2)	8835 (14.1)
Secondary+	24,295 (37.6)	33,989 (54.2)
**Partner’s Education status**		
No education	17,218 (26.7)	11,416 (18.3)
Primary	10,539 (16.3)	9407 (15)
Secondary+	36,850 (57)	41,731 (66.7)
**Wealth Status**		
Poorest	12,197 (18.9)	10,586 (16.9)
Poorer	12,847 (19.9)	12,065 (19.2)
Middle	12,867 (19.9)	12,864 (20.5)
Richer	13,116 (20.3)	13,337 (21.3)
Richest	13,580 (21)	13,864 (22.1)
**Religion**		
Hindu	52,569 (81.4)	50,856 (81.1)
Others	12,038 (18.6)	11,859 (18.9)
**Place of residence**		
Urban	19,808 (30.7)	22,070 (35.2)
Rural	44,799 (69.3)	40,646 (64.8)
**Region**		
North	8127 (12.6)	8050 (12.8)
Central	15,829 (24.5)	13,900 (22.2)
East	14,964 (23.2)	14,107 (22.5)
North-east	2235 (3.5)	2114 (3.4)
West	9463 (14.7)	9735 (15.5)
South	13,990 (21.7)	14,810 (23.6)
**Parity**		
0	6563 (10.2)	6072 (9.7)
1–2	25,772 (39.9)	32,119 (51.2)
3+	32,272 (50)	24,525 (39.1)
**Economic empowerment status**		
Not empower	–	27,743 (44.2)
Empower	–	34,973 (55.8)
**Witnessing parental Violence**		
Yes	52,713 (81.6)	49,642 (79.2)
No	11,894 (18.4)	13,074 (20.9)
**Duration of cohabitation in years**		
0–4	11,844 (18.3)	11,913 (19.0)
5–9	12,823 (19.9)	11,260 (18.0)
10–14	11,277 (17.5)	10,493 (16.7)
15–19	10,352 (16)	10,071 (16.1)
20+	18,311 (28.3)	18,980 (30.3)
**Number of co wives**		
None	63,513 (98.3)	61,733 (98.0)
One and more	1094 (1.7)	983 (2.0)
**Age at first marriage**		
Below 18	38,301 (59.3)	27,040 (43.1)
More than 18 years	26,306 (40.7)	35,676 (56.9)
**Partners controlling behaviours**		
Yes	38,833 (60.1)	33,763 (53.8)
No	25,774 (39.9)	28,953 (46.2)
**Partner drink alcohol**		
Yes	43,948 (68)	45,153 (72.0)
No	20,659 (32)	17,563 (28.0)

**Fig. 1:**
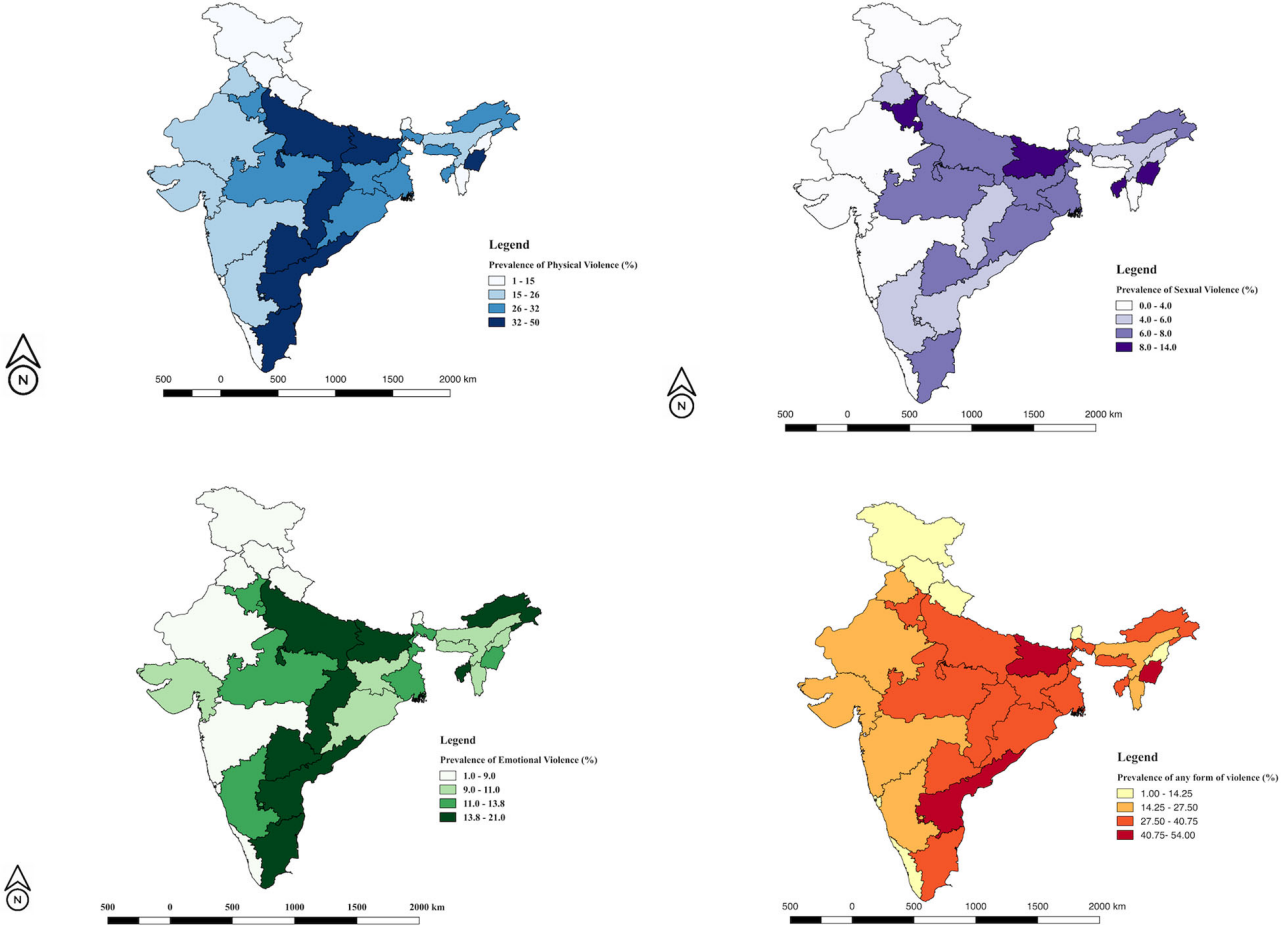
**a**-**d** State-wise variations in the prevalence of physical, sexual, emotional and anyform of IPV among the married women as per the fourth round of the National Family Health Survey, India (2015–16)

Physical violence was the most common form of IPV (29.2%) experienced by the respondents. Over all, the proportion of the respondents who ever experienced physical form of IPV (Table 2) decreased from round 3 to round 4 (Relative change of − 14.9%). The physical form of IPV continues to be reported from the highest age groups, uneducated, poorest respondents from Eastern part of India, who were mostly multiparous, married at a young age and were economically empowered. Also, the weighted prevalence was high among the respondents whose partners either had a controlling behavior, or were addicted to alcohol. Only southern region of the country depicted a relative increase in prevalence of IPV in round 4 compared to round 3. State-wise, maximum prevalence was observed in Manipur (50%), while minimum prevalence was seen in Sikkim (1%) as per the NFHS Round 4 (Supplementary Table 1; Fig. 1a). Multiple binary logistic regression analysis, depicted all the variables depicted in Table 1 as the significant predictor of physical form of domestic violence as per NFHS-4 datasets except older age groups, and less years of education.

**Table 2:** Percentage changes and correlates of physical IPV from 2005-06 to 2015-16 as per the National Family Health Survey (Rounds 3 and 4), India

	NFHS-3	NFHS-4	% Relative change	Unadjusted OR (95% CI)	Adjusted OR (95% CI) and ***p***-value	
**Total**	34.3 (33.9–34.6)	29.2 (28.5–29.8)	−14.9			
**Age**						
15–19 (Ref)	24.6 (23.4–25.8)	17.7 (16.1–19.2)	−28.0	1	1	
20–24	31.9 (31.1–32.8)	24.8 (23.9–25.6)	−22.3	1.4 (1.2–1.6)	1.2 (1.03–1.4)	0.02
25–34	35.1 (34.3–35.9)	28.9 (28.1–29.7)	− 17.7	1.5 (1.3–1.7)	1.0 (0.9–1.2)	0.77
35+	36 (35.5–36.5)	31.1 (30.6–31.5)	−13.6	1.7 (1.5–1.9)	1.0 (0.8–1.2)	0.94
**Respondents education**						
No education (Ref)	44.3 (43.7–44.8)	39.5 (38.7–40.2)	−10.8	1	1	
Primary	36.3 (35.4–37.3)	35.6 (34.6–36.6)	−1.9	0.8 (0.8–0.9)	1.0 (0.9–1.1)	0.82
Secondary+	20.9 (20.3–21.4)	21.5 (21.0–21.9)	2.9	0.4 (0.4–0.5)	0.8 (0.7–0.8)	0.00
**Partner Education**						
No education (Ref)	45.1 (44.3–45.8)	41.2 (40.3–42.1)	−8.6	1	1	
Primary	42.1 (41.1–43.0)	36.3 (35.3–37.3)	−13.8	0.9 (0.8–0.9)	1.0 (0.9–1.1)	0.96
Secondary+	27 (26.5–27.4)	24.3 (23.9–24.7)	−10.0	0.5 (0.5–0.5)	0.9 (0.8–0.9)	0.00
**Wealth Status**						
Poorest (Ref)	46.4 (45.5–47.3)	41.7 (40.8–42.7)	−10.1	1	1	
Poorer	43.2 (42.4–44.1)	36.4 (35.5–37.2)	−15.7	0.7 (0.7–0.7)	0.9 (0.9–1.0)	0.07
Middle	37.3 (36.5–38.2)	30.7 (29.9–31.5)	−17.7	0.5 (0.5–0.6)	0.8 (0.7–0.9)	0.00
Richer	29.8 (29.0–30.6)	25.2 (24.4–25.9)	−15.4	0.4 (0.4–0.4)	0.7 (0.6–0.7)	0.00
Richest	16.3 (15.6–16.9)	15.8 (15.2–16.4)	−3.1	0.3 (0.2–0.3)	0.5 (0.5–0.6)	0.00
**Religion**						
Hindu	34 (33.6–34.5)	30.2 (29.8–30.6)	−11.2	1	1	
Non-Hindu	35.2 (34.4–36.1)	27.1 (26.3–27.9)	−23.0	0.7 (0.7–0.7)	0.9 (0.9–0.9)	0.00
**Place of residence**						
Urban	27.8 (27.2–28.5)	23.5 (23.0–24.1)	−15.5	1	1	
Rural	37.1 (36.6–37.5)	32.2 (31.7–32.6)	−13.2	**1.4 (1.3–1.4)**	**0.9 (0.8–0.9)**	0.00
**Region**						
North(Ref)	29.0 (28.0–30.0)	21 (20.06–21.8)	−27.6	1	1	
Central	40.2 (39.5–41.0)	33.2 (32.5–34.0)	−17.4	2.1 (2.0–2.2)	1.3 (1.3–1.4)	0.00
East	40.7 (39.9–41.5)	34.8 (34.0–35.5)	−14.5	2.5 (2.4–2.7)	1.4 (1.3–1.5)	0.00
North-east	33.1 (31.1–35.0)	23.7 (21.9–25.5)	−28.4	1.4 (1.3–1.5)	1.1 (1.0–1.2)	0.01
West	27.6 (26.7–28.5)	19.5 (18.7–20.2)	−29.3	1.1 (1.0–1.1)	1.1 (1.0–1.2)	0.08
South	28.3 (27.6–29.1)	31.7 (31.0–32.5)	12.0	1.9 (1.8–2.0)	1.4 (1.3–1.5)	0.00
**Parity**						
0(Ref)	21.4 (20.4–22.4)	16.1 (15.2–17.0)	−24.8	1		
1–2	27.5 (27.0–28.1)	25.9 (25.5–26.4)	−5.8	1.5 (1.4–1.7)	1.5 (1.3–1.6)	0.00
3+	42.2 (41.7–42.8)	36.7 (36.1–37.3)	−13.0	2.5 (2.3–2.7)	1.7 (1.6–1.9)	0.00
**Economic empowerment status**						
Not empower	–	25.7 (25.1–26.2)	–	1	1	
Empower	–	32.0 (31.5–32.5)	–	**1.4 (1.3–1.4)**	**1.1 (1.0–1.1)**	0.00
**Witnessing parental Violence**						
No(Ref)	29.3 (28.9–29.7)	23.0 (22.6–23.3)	−21.5	1		
Yes	56.4 (55.5–57.2)	52.8 (51.9–53.6)	−6.4	4.1 (3.9–4.3)	3.3 (3.2–3.5)	0.00
**Duration of cohabitation in years**						
0–4(Ref)	20.9 (20.2–21.7)	17.6 (16.9–18.3)	−15.8	1	1	
5–9	34.1 (33.3–34.9)	29.6 (28.7–30.4)	− 13.2	1.7 (1.6–1.8)	1.4 (1.3–1.6)	0.00
10–14	38.7 (37.8–39.6)	31.8 (30.9–32.7)	−17.8	1.9 (1.8–2.1)	1.6 (1.4–1.7)	0.00
15–19	37.9 (37.0–38.9)	32.4 (31.5–33.3)	−14.5	2.0 (1.8–2.1)	1.5 (1.4–1.7)	0.00
20+	38.2 (37.5–38.9)	33.0 (32.4–33.7)	−13.6	2.0 (1.9–2.1)	1.5 (1.4–1.7)	0.00
**Number of co wives**						
None(Ref)	34 (33.6–34.4)	28.8 (28.5–29.2)	−15.3	1	1	
One and more	48.6 (45.6–51.5)	51.5 (48.4–54.6)	6.0	1.9 (1.7–2.2)	1.6 (1.3–1.8)	0.00
**Age at first marriage**						
Below 18(Ref)	40.1 (39.6–40.6)	34.6 (34.1–35.2)	−13.7	1		
More than 18 years	25.7 (25.2–26.3)	25.1 (24.6–25.5)	−2.3	0.6 (0.6–0.7)	0.9 (0.9–0.9)	0.00
**Partners controlling behaviours**						
No(Ref)	24.1 (23.6–24.5)	17.9 (17.5–18.3)	−25.7	1	1	
Yes	49.6 (49.0–50.2)	42.3 (41.7–42.9)	−14.7	3.4 (3.3–3.6)	2.8 (2.7–2.9)	0.00
**Partner drink alcohol**						
No(Ref)	27 (26.6–27.4)	21.2 (20.8–21.5)	−21.5	1	1	
Yes	49.7 (49.0–50.3)	49.8 (49.1–50.5)	0.2	3.4 (3.3–3.5)	2.6 (2.5–2.7)	0.00

Sexual form depicted minimum weighted prevalence amongst all types of IPV, and decreased (− 30.2%), from round 3 (9.6%) to round 4 (6.7%) (Table 3). The weighted prevalence as per the round 4 of NFHS is maximum in the 25–34 years age group, uneducated, poorest respondents from Eastern part of the country who were married at a younger age, were multiparous, and economically empowered. State wise weighted prevalence of Sexual IPV ranged between (14% in Bihar and Manipur to 0% in Sikkim) (Supplementary Table 1; Fig. 1a). Similar to physical form of IPV, weighted prevalence was more among the respondents, whose partners had a controlling behavior, or were consuming alcohol, and had increased in Southern region of the country. Binary logistic regression depicted all the independent variables as significant predictors of sexual IPV except age, less years of education of respondents and their partners, parity, more than 20 years of cohabitation, and age of marriage of the respondents.

**Table 3:** Percentage changes and correlates of sexual IPV from 2005-06 to 2015–16 as per the National Family Health Survey (Rounds 3 and 4), India

	NFHS-3	NFHS-4	% Relative change	Unadjusted OR (95% CI)	Adjusted OR (95% CI) and ***p***-Value	
**Total**	9.6 (9.40–9.86)	6.7 (5.9–7.4)	−30.2			
**Age**						
15–19 (Ref)	12.3 (11.4–13.3)	5.6 (4.7–6.6)	−54.5	1	1	
20–24	10.2 (9.7–10.8)	6.3 (5.8–6.7)	−38.2	1.1 (0.9–1.3)	1.1 (0.8–1.4)	0.67
25–34	9.9 (9.4–10.4)	7.0 (6.6–7.5)	−29.3	1.1 (0.9–1.3)	0.9 (0.7–1.2)	0.58
35+	9.0 (8.7–9.3)	6.7 (6.4–6.9)	−25.6	1.0 (0.8–1.3)	0.9 (0.7–1.2)	0.35
**Respondents education**						
No education (Ref)	12.3 (11.9–12.6)	9.2 (8.8–9.6)	−25.2	1	1	
Primary	10.3 (9.7–10.9)	8.1 (7.5–8.6)	−21.4	0.9 (0.8–1.0)	1.1 (1.0–1.2)	0.30
Secondary+	6.1 (5.8–6.6)	4.8 (4.6–5.0)	−21.3	0.5 (0.5–0.6)	0.9 (0.8–1.0)	0.10
**Partner Education**						
No education (Ref)	12.7 (12.2–13.2)	9.9 (9.4–10.5)	−22.0	1	1	
Primary	11.7 (11.1–12.3)	8.6 (8.1–9.2)	−26.5	0.9 (0.8–1.0)	1 (0.9–1.1)	0.94
Secondary+	7.6 (7.3–7.9)	5.3 (5.1–5.5)	−30.3	0.5 (0.5–0.6)	0.9 (0.8–0.9)	0.02
**Wealth Status**						
Poorest (Ref)	14.3 (13.64–14.88)	11.6 (10.9–12.1)	−18.9	1	1	
Poorer	12.2 (11.59–12.73)	8.2 (7.7–8.6)	−32.8	0.7 (0.6–0.7)	0.9 (0.82–0.99)	0.03
Middle	10.2 (9.65–10.69)	6.7 (6.2–7.1)	−34.3	0.6 (0.5–0.6)	0.9 (0.82–1.02)	0.08
Richer	7.9 (7.40–8.32)	5 (4.6–5.4)	−36.7	0.4 (0.4–0.5)	0.8 (0.69–0.89)	0.00
Richest	4.3 (39.46–41.10)	3.2 (2.9–3.5)	−25.6	0.3 (0.2–0.3)	0.6 (0.55–0.74)	0.00
**Religion**						
Hindu	9.3 (9.08–9.58)	6.6 (6.4–6.8)	−29.0	1	1	
Non-Hindu	11 (10.39–11.51)	6.8 (6.3–7.2)	−38.2	0.9 (0.8–0.9)	1.1 (1.00–1.19)	0.05
**Place of residence**						
Urban	6.8 (6.45–7.15)	4.8 (4.49–5.05)	−29.4	**1**	**1**	
Rural	10.9 (10.59–11.17)	7.6 (7.37–7.89)	−30.3	**1.4 (1.3–1.5)**	**0.9 (0.86–1.03)**	0.19
**Region**						
North(Ref)	11.9 (11.21–12.61)	4.6 (4.1–5.0)	−61.3	1	1	
Central	9.1 (8.69–9.59)	7.4 (6.9–7.8)	−18.7	1.7 (1.5–1.9)	1.1 (0.94–1.18)	0.28
East	18.4 (17.74–18.98)	10 (9.5–10.5)	−45.7	2.6 (2.3–2.8)	1.4 (1.25–1.59)	0.00
North-east	12.6 (11.19–13.93)	5.6 (4.6–6.6)	−55.6	1.5 (1.3–1.7)	1.2 (1.05–1.39)	0.01
West	3.5 (3.12–3.86)	2.8 (2.5–3.1)	−20.0	0.8 (0.7–0.9)	0.8 (0.70–0.99)	0.03
South	3.2 (2.91–3.49)	6.6 (6.2–7)	106.3	1.4 (1.3–1.6)	1.0 (0.87–1.14)	1.00
**Parity**						
0(Ref)	9 (8.35–9.73)	4.8 (4.3–5.3)	−46.7	1	1	
1–2	8.1 (7.74–8.40)	5.6 (5.3–5.8)	−30.9	1.0 (0.9–1.2)	1.0 (0.89–1.21)	0.57
3+	11 (10.65–11.33)	8.5 (8.2–8.9)	−22.7	1.5 (1.4–1.7)	1.2 (1.04–1.44)	0.02
**Economic empowerment status**						
Not empower	–	5.9 (5.6–6.2)	–	1	1	
Empower	–	7.3 (7.0–7.5)	–	1.3 (1.2–1.4)	1.1 (1.1–1.2)	0.01
**Witnessing parental Violence**						
No(Ref)	8 (7.79–8.25)	4.9 (4.7–5.1)	−38.8	1	1	
Yes	16.8 (16.09–17.43)	13.3 (12.7–13.8)	−20.8	3.1 (2.9–3.3)	2.2 (2.0–2.4)	0.00
**Duration of cohabitation in years**						
0–4(Ref)	8.7 (8.14–9.16)	4.7 (4.3–5.0)	−46.0	1	1	
5–9	10.1 (9.62–10.66)	7.2 (6.7–7.7)	−28.7	1.4 (1.2–1.5)	1.2 (1.06–1.41)	0.01
10–14	10.7 (10.15–11.29)	7.4 (6.9–7.9)	−30.8	1.5 (1.3–1.6)	1.3 (1.09–1.53)	0.00
15–19	9.8 (9.20–10.34)	7.3 (6.8–7.8)	−25.5	1.5 (1.4–1.7)	1.3 (1.06–1.57)	0.01
20+	9.2 (8.73–9.57)	6.8 (6.5–7.2)	−26.1	1.3 (1.2–1.5)	1.1 (0.93–1.39)	0.20
**Number of co wives**						
None(Ref)	9.5 (9.30–9.76)	6.5 (6.3–6.7)	−31.6	1	11	
One and more	15.2 (13.11–17.37)	17.4 (15.0–19.7)	14.5	2.3 (1.9–2.8)	1.9 (1.55–2.28)	0.00
**Age at first marriage**						
Below 18(Ref)	11.7 (11.38–12.02)	7.8 (7.5–8.2)	−33.3	1	1	
More than 18 years	6.6 (6.32–6.92)	5.8 (5.5–6.0)	−12.1	0.7 (0.6–0.7)	0.9 (0.87–1.01)	0.12
**Partners controlling behaviours**						
No(Ref)	5.6 (4.79–5.23)	2.2 (2.0–2.3)	−60.7	1	1	
Yes	16.6 (16.14–17.04)	11.9 (11.5–12.3)	−28.3	5.5 (5.1–5.9)	4.3 (3.95–4.66)	0.00
**Partner drink alcohol**						
No(Ref)	7.6 (7.34–7.84)	4 (3.8–4.2)	−47.4	1	1	
Yes	14 (13.49–14.43)	13.4 (12.9–13.9)	− 4.3	3.3 (3.1–3.6)	2.4 (2.21–2.54)	0.00

Emotional violence was more common than sexual violence but less than the physical form with a national prevalence of around 13% (Table 4). The weighted prevalence was nearly similar in all age groups as per NFHS 4, but highest in uneducated, and poorest quintile of respondents belonging to the eastern region of India, who were multiparous and economically empowered similar to other forms of violence. State wise weighted prevalence of emotional IPV ranged between (21% in Tamil Nadu to 1% in Sikkim) (Supplementary Table 1; Fig. 1c). More number of co-wives, early age of marriage, controlling behavior of partners, and alcohol also increased weighted prevalence of emotional form of IPV. Binary logistic regression depicted that there were higher odds of experiencing emotional violence when the respondents were young, uneducated, belong to the poorest quintile, and southern region of India, multiparity, history of witnessing parental violence, more duration of cohabitation after marriage, controlling behaviours of the partner, and alcohol consumptions.

**Table 4:** Percentage changes and correlates of emotional IPV from 2005-06 to 2015-16 as per the. National Family Health Survey (Rounds 3 and 4), India

	NFHS 3	NFHS 4	% Relative change	Unadjusted OR (95% CI)	Adjusted OR (95% CI) and ***p***-Value	
**Total**	14.8 (14.5–15.1)	13.2 (12.4–13.8)	−11.0			
**Age**						
15–19 (Ref)	12.0 (11.0–12.9)	12.7 (10.7–13.4)	0.7	1	1	
20–24	13.4 (12.8–14.2)	11.2 (10.5–11.8)	−16.8	1.0 (0.8–1.1)	0.8 (0.7–0.9)	0.03
25–34	15.0 (14.4–15.6)	12.6 (12.0–13.2)	−16	1.1 (0.9–1.2)	0.8 (0.6–0.9)	0.01
35+	15.6 (15.2–15.9)	13.9 (13.6–14.3)	−10.5	1.1 (1.0–1.3)	0.7 (0.6–0.9)	0.00
**Respondents education**						
No education (Ref)	18.4 (18.0–18.9)	17.8 (17.3–18.4)	−3.2	1	1	
Primary	16.1 (15.4–16.9)	15.0 (14.2–15.7)	−7.3	0.8 (0.8–0.9)	1.0 (0.9–1.0)	0.36
Secondary+	9.7 (9.3–10.1)	10.0 (9.6–10.3)	2.6	0.5 (0.5–0.6)	0.9 (0.8–0.9)	0.00
**Partner Education**						
No education (Ref)	19.5 (18.9–20.1)	19.1 (18.4–19.9)	−1.9	1	1	
Primary	18.2 (17.4–18.9)	15.6 (14.8–16.3)	−14.4	0.8 (0.8–0.9)	0.9 (0.9–1.0)	0.17
Secondary+	11.6 (11.3–12.0)	11 (10.7–11.3)	−5.6	0.6 (0.5–0.6)	0.9 (0.8–0.9)	0.00
**Wealth Status**						
Poorest (Ref)	19.6 (18.9–20.3)	18.5 (17.7–19.2)	−5.8	1	1	
Poorer	18.9 (18.2–19.6)	16.0 (15.3–16.6)	−15.5	0.8 (0.8–0.9)	0.9 (0.9–1.0)	0.09
Middle	16.2 (15.6–16.9)	14.1 (13.5–14.7)	−13	0.7 (0.6–0.7)	0.8 (0.8–0.9)	0.00
Richer	12.0 (11.5–12.6)	11.2 (10.7–11.8)	−6.6	0.5 (0.5–0.6)	0.7 (0.6–0.7)	0.00
Richest	7.9 (7.4–8.3)	7.6 (7.2–8.1)	−3.4	0.4 (0.3–0.4)	0.6 (0.5–0.6)	0.00
**Religion**						
Hindu	14.8 (14.5–15.2)	13.4 (13.1–13.7)	−9.6	1	1	
Non-Hindu	14.6 (14.0–15.3)	12.8 (12.2–13.4)	−12.8	**0.9 (0.8–0.9)**	**1.2 (1.1–1.2)**	0.00
**Place of residence**						
Urban	12.0 (11.5–12.4)	11.2 (10.8–11.6)	−6.8	1	1	
Rural	16.0 (15.7–16.4)	14.2 (13.9–14.5)	−11.4	1.2 (1.1–1.3)	0.8 (0.8–0.9)	0.00
**Region**						
North(Ref)	14.4 (13.7–15.2)	8.9 (8.3–9.5)	−38.5	1	1	
Central	16.6 (16.0–17.2)	12.9 (12.3–13.4)	−22.4	1.5 (1.4–1.6)	0.9 (0.8–1.3)	0.05
East	16.0 (15.4–16.6)	14.8 (14.2–15.4)	−7.2	1.8 (1.7–1.9)	1 (0.9–1.0)	0.29
North-east	14.0 (12.6–15.5)	10.7 (9.4–12.0)	−23.5	1.3 (1.2–1.4)	1.1 (1.0–1.2)	0.17
West	16.4 (15.7–17.2)	9.6 (95.2–96.0)	−41.7	1.2 (1.0–1.3)	1.2 (1.1–1.4)	0.00
South	10.7 (10.2–11.2)	16.9 (16.3–17.5)	57.6	2.0 (1.9–2.2)	1.6 (1.4–1.7)	0.00
**Parity**						
0(Ref)	10.5 (9.8–11.3)	9.3 (92.5–93.7)	−11.5	1	1	
1–2	12.5 (12.1–12.9)	11.8 (11.4–12.1)	−5.8	1.3 (1.2–1.4)	1.3 (1.1–1.4)	0.00
3+	17.5 (17.1–17.9)	15.9 (15.4–16.4)	−9	1.8 (1.7–2.0)	1.4 (1.3–1.6)	0.00
**Economic empowerment status**						
Not empower	–	11.5 (11.1–11.9)	11.5	1	1	
Empower	–	14.5 (14.1–14.9)	14.5	1.0 (0.9–1.1)	1.0 (0.9–1.1)	0.68
**Witnessing parental Violence**						
No(Ref)	12 (11.7–12.3)	10.1 (9.8–10.4)	−15.9	1	1	
Yes	27.2 (26.4–27.9)	24.8 (24.1–25.5)	−8.7	3.1 (2.9–3.3)	2.2 (2.1–2.3)	0.00
**Duration of cohabitation in years**						
0–4(Ref)	10.1 (9.6–10.6)	9 (8.5–9.5)	−10.6	1	1	
5–9	14.5 (13.9–15.1)	12.8 (12.2–13.4)	−11.8	1.4 (1.3–1.5)	1.3 (1.1–1.4)	0.00
10–14	15.9 (15.2–16.5)	14 (13.4–14.7)	−11.5	1.6 (1.4–1.7)	1.4 (1.2–1.6)	0.00
15–19	16.5 (15.8–17.2)	14.4 (13.7–15.1)	−12.8	1.6 (1.5–1.8)	1.4 (1.2–1.6)	0.00
20+	16.4 (15.9–17.0)	14.9 (14.3–15.4)	−9.6	1.7 (1.6–1.8)	1.5 (1.3–1.7)	0.00
**Number of co wives**						
None(Ref)	14.6 (14.3–14.9)	12.9 (12.6–13.2)	−11.6	1	1	
One and more	26.4 (23.7–29.0)	29.3 (26.5–32.2)	11.3	2.3 (2.0–2.7)	1.9 (1.6–2.2)	0.00
**Age at first marriage**						
Below 18(Ref)	17.2 (16.8–17.6)	15.3 (14.9–15.7)	−10.9	1	1	
More than 18 years	11.3 (10.9–11.7)	11.5 (11.2–11.9)	2	0.7 (0.7–0.8)	1.0 (0.9–1.1)	0.72
**Partners controlling behaviours**						
No(Ref)	7.5 (7.3–7.8)	5.3 (5.1–5.5)	−29.5	1	1	
Yes	25.7 (25.2–26.3)	22.3 (21.8–22.8)	−13.3	5.1 (4.8–5.4)	4.5 (4.2–4.7)	0.00
**Partner drink alcohol**						
No(Ref)	11.3 (11.0–11.6)	9.0 (8.7–9.3)	−20.2	1	1	
Yes	22.3 (21.7–22.9)	23.9 (23.2–24.5)	7	3.0 (2.8–3.1)	2.2 (2.1–2.3)	0.00

The weighted prevalence of any type of IPV ever faced by a woman decreased from 38.9% in third round to 32.8% in the fourth round. Maximum relative % decrease was seen in youngest age group, uneducated (− 10.9%), middle quintile (− 17.4%), Northern region (− 30.9%), nulliparous female respondents (− 26.4%), amongst those who did not witness any kind of parental violence (− 22.7%), who were coinhabiting for less than 5 years (− 21.5%), and were not living with any co-wives of their partners (− 16.5%). State wise weighted prevalence of any type of IPV ranged between (56% in Manipur to 2% in Sikkim) (Supplementary Table 1; Fig. 1d). Significant predictors of any type of IPV as per the fourth round of NFHS are depicted in Table 5. It was seen that economic empowerment of women, and age at first marriage could not predict exposure to IPV among the respondents.

**Table 5:** Percentage changes and correlates of any form of IPV  from 2005-06 to 2015-16 as per the National Family Health Survey (Rounds 3 and 4), India

	NFHS 3	NFHS 4	% Relative change	Unadjusted OR (95% CI)	Adjusted OR (95% CI) and ***p***-value	
**Total**	38.9 (38.55–39.31)	32.8 (32.4–33.2)	−15.7			
**Age**						
15–19 (Ref)	33.0 (31.7–34.4)	23.1 (21.4–24.9)	−30.0	1	1	
20–24	37.0 (36.1–37.8)	28.4 (27.5–29.3)	−23.2	1.3 (1.2–1.5)	1.1 (0.9–1.3)	0.07
25–34	39.4 (38.5–40.2)	32.3 (31.5–33.1)	−18.0	1.4 (1.3–1.6)	1.0 (0.8–1.2)	0.82
35+	40.2 (39.7–40.7)	34.6 (34.1–35.0)	−13.9	1.5 (1.4–1.7)	0.9 (0.8–1.1)	0.44
**Respondents education**						
No education (Ref)	48.8 (48.3–49.4)	43.5 (42.8–44.2)	−10.9	1	1	
Primary	41.6 (40.6–42.5)	39.0 (37.9–40.0)	−6.3	0.8 (0.8–0.9)	1 (0.9–1.0)	0.38
Secondary+	25.5 (24.9–26.0)	24.8 (24.4–25.3)	−2.7	0.5 (0.4–0.5)	0.8 (0.7–0.8)	0.00
**Partner Education**						
No education (Ref)	49.7 (48.9–50.4)	45.1 (44.2–46.0)	−9.3	1	1	
Primary	46.6 (45.7–47.6)	40 (39.0–41.0)	−14.2	0.9 (0.8–0.9)	1 (0.9–1.1)	0.67
Secondary+	31.7 (31.2–32.2)	27.7 (27.3–28.1)	−12.6	0.5 (0.5–0.5)	0.9 (0.8–0.9)	0.00
**Wealth Status**						
Poorest (Ref)	51.8 (50.9–52.6)	45.5 (44.6–46.5)	−12.2	1	1	
Poorer	48.5 (47.6–49.3)	40.3 (39.4–41.2)	−16.9	0.7 (0.7–0.8)	0.9 (0.9–1.0)	0.05
Middle	41.9 (41.0–42.7)	34.6 (33.8–35.5)	−17.4	0.6 (0.5–0.6)	0.8 (0.8–0.9)	0.00
Richer	34.2 (33.4–35.0)	28.5 (27.7–29.3)	−16.7	0.4 (0.4–0.5)	0.7 (0.7–0.8)	0.00
Richest	20.2 (19.5–20.9)	18.8 (18.1–19.4)	−6.9	0.3 (0.3–0.3)	0.5 (0.5–0.6)	0.00
**Religion**						
Hindu	38.8 (38.4–39.2)	33.7 (33.2–34.1)	−13.1	1	1	
Others	39.5 (38.6–40.3)	30.9 (30.1–31.7)	−21.8	0.8 (0.7–0.8)	1 (0.9–1.0)	0.25
**Place of residence**						
Urban	31.4 (30.7–32.0)	24.4 (23.8–25.0)	−22.3	1	1	
Rural	42.3 (41.8–42.7)	33.5 (33.1–34.0)	−20.8	1.4 (1.3–1.4)	0.9 (0.9–0.9)	0.00
**Region**						
North(Ref)	34.6 (33.5–35.6)	23.9 (23.0–24.9)	−30.9	1	1	
Central	44.3 (43.5–45.1)	36.4 (35.6–37.2)	−17.8	2.0 (1.9–2.1)	1.3 (1.3–1.5)	0.00
East	47.9 (47.1–48.7)	38.6 (37.8–39.4)	−19.4	2.4 (2.2–2.5)	1.3 (1.4–1.6)	0.00
North-east	38.3 (36.3–40.4)	27.1 (25.2–29.0)	−29.2	1.4 (1.4–1.5)	1.2 (1.1–1.2)	0.00
West	32.2 (31.2–33.1)	22 (21.2–22.8)	−31.7	1.0 (1.0–1.1)	1.1 (1.0–1.2)	0.06
South	30.5 (29.7–31.2)	32.8 (35.7–37.2)	7.5	1.9 (1.8–2.0)	1.4 (1.3–1.5)	0.00
**Parity**						
0(Ref)	27.6 (26.6–28.7)	20.3 (19.3–21.3)	−26.4	1	1	
1–2	32.6 (32.0–33.1)	29.2 (28.7–29.7)	−10.4	1.4 (1.3–1.5)	1.4 (1.3–1.5)	0.00
3+	46.3 (45.8–46.9)	40.4 (39.8–41.0)	−12.7	2.3 (2.1–2.4)	1.6 (1.5–1.8)	0.00
**Economic empowerment status**						
Not empower	–	29.1 (28.7–29.5)	–	1	1	
Empower	–	35.7 (34.8–36.5)	–	1.4 (1.3–1.4)	1.1 (1.1–1.1)	0.00
**Witnessing parental Violence**						
No(Ref)	33.9 (33.5–34.3)	26.2 (25.8–26.7)	−22.7	1	1	
Yes	61.1 (60.2–62.0)	57.5 (56.9–58.0)	−5.9	4.1 (4.0–4.3)	3.4 (3.2–3.5)	0.00
**Duration of cohabitation in years**						
0–4(Ref)	27.0 (26.2–27.8)	21.2 (20.5–21.9)	−21.5	1	1	
5–9	39.1 (38.3–39.9)	32.8 (32.0–33.7)	−16.1	1.6 (1.5–1.7)	1.4 (1.3–1.5)	0.00
10–14	42.8 (41.9–43.7)	35.3 (34.4–36.2)	−17.5	1.8 (1.7–1.9)	1.5 (1.4–1.7)	0.00
15–19	42.2 (41.2–43.1)	36.3 (35.4–37.3)	−14.0	1.9 (1.8–2.0)	1.5 (1.4–1.7)	0.00
20+	42.4 (41.6–43.1)	36.6 (36.0–37.3)	−13.7	1.9 (1.8–2.0)	1.5 (1.3–1.7)	0.00
**Number of co wives**						
None(Ref)	38.7 (38.3–39.1)	32.4 (32.0–32.7)	−16.3	1	1	
One and more	52.1 (49.1–55.0)	55.5 (52.4–58.6)	6.5	1.9 (1.7–2.2)	1.5 (1.3–1.8)	0.00
**Age at first marriage**						
Below 18(Ref)	45 (44.5–45.5)	38.4 (37.8–39.0)	−14.7	1	1	
More than 18 years	30.1 (29.5–30.6)	28.5 (28.0–28.9)	−5.3	0.6 (0.6–0.7)	0.9 (0.9–0.9)	0.00
**Partners controlling behaviours**						
No(Ref)	27.8 (27.4–28.2)	19.9 (19.5–20.4)	−28.4	1	1	
Yes	55.7 (54.5–57.0)	47.7 (47.1–48.3)	−14.4	3.7 (3.5–3.8)	3.2 (3.0–3.3)	0.00
**Partner drink alcohol**						
No(Ref)	32.0 (31.5–32.4)	32.4 (32.0–32.8)	1.3	1	1	
Yes	53.8 (53.1–54.5)	59.8 (59.0–60.5)	11.2	3.3 (3.2–3.4)	2.5 (2.4–2.6)	0.00

## Discussion

There are some major findings of our study. First, there was a decrease in any form of IPV from NFHS round 3 to 4. Maximum reduction was observed in sexual IPV, followed by physical and emotional form. But still, around 7 out of 100 women reported history of sexual IPV. Second, IPV was reported more amongst the poorest and uneducated respondents. Third, contrary to our belief, urban areas depicted a higher chance of IPV. Fourth, we observed that certain factors like economic empowerment that were considered to act as a shield against IPV were of no help, but increased the probability of violence episodes. Lastly, we observed that most of the factors predicting the exposure to different kinds of violence were same. Decrease in IPV in NFHS-4 can be attributed to improvement in education and societal status, decrease in witnessing family violence, improved sense of gender equality, more awareness among women, improved community norms regarding domestic violence, and enforcement of the PWDVA after 2006 .

We observed that sexual IPV has depicted maximum decrease in India as per the two rounds, compared to other forms of IPV. Coerced sex as seen in sexual violence may result in sexual gratification on the part of the perpetrator, though its underlying purpose is to dominate the spouse through force. Also, such men feel that their actions are in accordance with the law as they are married the victim. However, sexual violence has a profound impact on physical and mental health because it also leads to physical injuries, a plethora of acute and chronic sexual and reproductive health problems [36]. To our dismay, it is a neglected area of research, the available data is scanty and fragmented as many women do not report sexual violence due to emotional embarrassment, or fear of being blamed. Also, only a very small proportion of women seek medical services for immediate problems related to sexual violence.

We observed a higher incidence of IPV among the poorest and uneducated respondents [37]. It is well documented that people from the under-privileged sections of the society are at increased risk of IPV [38]. Low economic status also has many associated stressors like economic stress, that are linked with marital conflicts [39, 40]. According to the *family stress model*, lack of money or increased expenditure, induces frequent emotional outbursts and, conflicts among family members, including conflict between spouses [40]. Also, the women who is a victim to IPV, experience several negative outcomes like decreased economic productivity in addition to poor psychosomatic health as a vicious cycle.

We also observed a higher chance of having IPV in urban areas compared to rural areas. On bivariable logistic regression, there was higher chances of violence in rural areas, but this was reversed during the adjusted analysis. This contradictory pattern has also been noted in Bolivia, Haiti and Zambia, where women living in urban areas were more likely to report partner violence than women living in rural areas [41]. There are several factors that can explain such trend. Some men may find economically independent and educated female partners threatening. There is evidence that increase in women’s empowerment, abates men’s feelings of control over their spouses that leads to increased violence to exert their control and power [42]. Further, urban areas provide women with greater opportunities to report violence, contrary to the rural areas, where access to appropriate health care services including the counselling of the victims and management of IPV injuries is more limited [43]. Also, interpersonal relations are more compromised and strenuous in urban areas due to pressures of urban living, such as poverty, engagement in certain types of occupation, poor quality living conditions and the physical configuration of urban areas, which can lead to greater incidence of violence [44]. On the other hand, rural areas in India depict better social support compared to the urban [45]. However, some studies depict higher chances of IPV in rural areas and it attributed to patriarchal ideology, and traditional gender roles [46]. Women with more children tend to be a higher risk of IPV similar to our observations and it was more in urban areas compared to rural [46]. This may be indirectly linked to increased economic stress in families with more children.

Economic empowerment was not seen to be protective against IPV in our study. Similar observations were reported in various sub-national analysis from India and abroad [47–51]. A longitudinal study of married women in Bangalore found that women who were unemployed but began employment subsequently had an 80% higher odds of violence, as compared to women who maintained their unemployed status [49]. Another study for a violence against women and girls reduction programme in India found that women who earned and controlled their own income were more likely to report violence experienced both at home [50]. One of the study also reports that till the time women’s income is less than her male spouse, empowerment is protective, and as the scenario changes with increase in her income, violence increase [51]. This increase in risk is related to ‘male backlash’ – as women gain more economic autonomy, men feel that their authority is being challenged and thus increase their use of violence as a means of reasserting their control [49, 51]. It is hypothesized that in less developed settings, where women are not independent economically, their entry into work may initially increase marital tensions and risk of IPV, but the tussle gradually settles down as their male counterparts start recognizing the benefits of additional household income [52]. This theory is supported by cross-country analysis [53]. However, the relationship between economic empowerment and violence is not universally the same. Studies from our neighbouring countries like Pakistan, Nepal, and Bangladesh depicted that the lifetime experience of IPV was high among the women with low empowerment [54, 55]. Another study from Jordan reported that the women who can take decision independently in the household matters and income related issues are less likely to suffer from IPV [56]. The relationship between women empowerment and violence is complex, and hence further investigation is required to understand which factors drive such findings in Indian context.

As noted, acceptability of IPV remains high in India, and in fact have seen little changes between the last two rounds of NFHS [10]. Furthermore, the impact of parental violence on their children was highlighted through our study. Families where parental violence was witnessed, or husbands exerted a controlling behavior depicted a higher risk of IPV [57]. Unfortunately, each form of family violence begets interrelated forms of violence, and the “cycle of abuse” is often continued from exposed children into their adult relationships, and finally to the care of the elderly [57]. Mothers should encourage daughters to engage in a relationship with responsible men, while fathers’ communication should be directed towards young boys and aimed at inculcating values against dominant traditional masculinity, objectifying girls and chauvinist values.

There are certain strength and limitations of this study that should be acknowledged. The study was done using the national data collected following a robust methodology that increases the reliability of data generated. The use of complex weighted analytical design to obtain results allow us to generalize the results for projection at national level. However, the use of secondary data in itself is a limitation of the study, as the results will contain only the predetermined variables. There are a lot more number of variables apart from those included in the NFHS that affect IPV and its effects on a female experiencing it. Lastly, if the data is self-reported, the overall true prevalence of the violence may actually be higher than estimated, owing to various reasons discussed earlier in the manuscript.

There are a few policy implications of this study. Given a high prevalence of physical and mental health problems in women exposed to IPV, there is a potential for cognitive behavioral interventions to improve women’s mental health. Advocacy related to IPV is necessary. It should be implemented through a multifaceted approach in the form of legal, housing and financial advices. Awareness should be aimed about the access to existing community resources such as shelters, hostels and psychological interventions and provision of legal support. Informal counselling is another intervention that may be offered to women [58]. However evidence from a Cochrane review regarding the effect of advocacy for women exposed to IPV has been equivocal [58]. Therefore, the future research should try to look for the best options that can be offered to such women who are seeking help. Also, the role of screening for IPV has been debated over recent years. The routine screening of women for IPV in health settings, in the absence of structured intervention, was to have limited impact upon health outcomes and re-exposure to violence and hence not recommended [18, 59]. However, some other studies from different study setting are in favor of offering screening services, and hence this also needs to be evaluated in different socio-cultural settings [60]. Finally, exposure to violence has significant impacts. Longitudinal studies are needed to understand the temporal relationship between recent IPV and different health issues, while considering the differential effects of recent versus past exposure to IPV [60]. Healthcare providers and IPV organizations should be aware of the bidirectional relationship between recent IPV and psycho-somatic symptoms. This will also improve our understanding of the immediate and long-term health needs of women exposed to IPV.

## Conclusions

We observed different patterns of IPV and their risk factors through this comprehensive assessment, and concluded that much needs to done in this regards. ﻿Our concepts of decreasing IPV through empowerment of women has been challenged, and so it the urban-rural divide. We must understand that in order to decrease IPV, we need to think beyond women, and focus more on challenges emerging from increase in women’s empowerment, and increase in urbanization. Interventions to empower women must work with couple as a unit, and at the community-level, to address equal job opportunities, and gender specific roles.

## Supplementary Information

**Additional file 1: Supplementary Table 1.** State wise trends in the different types of Intimate partner violence as per NFHS Round 3 & 4.

## Data Availability

This study analyses a nationally representative survey database which is available freely in public domain.
