# Supplementary material for: Trends and correlates of intimate partner violence experienced by ever-married women of India: results from National Family Health Survey round III and IV
Source: BMC Public Health. 2021 Nov 5;21:2012. doi: 10.1186/s12889-021-12028-5 (PMC8570022; doi:10.1186/s12889-021-12028-5)
Supplement: Supplementary file 1 — Additional file 1: Supplementary Table 1. State wise trends in the different types of Intimate partner violence as per NFHS Round 3 & 4. [file 12889_2021_12028_MOESM1_ESM.docx]

**Supplementary Table 1: State wise trends in the different types of Intimate partner violence as per NFHS Round 3&4.**

|  | | **Physical Violence** | | | **Sexual Violence** | | | **Emotional Violence** | | | **Any kind of violence** | | | |
| --- | --- | --- | --- | --- | --- | --- | --- | --- | --- | --- | --- | --- | --- | --- |
|  |  | NFHS-3 | NFHS-4 | % Diff | NFHS-3 | NFHS-4 | % Diff | NFHS-3 | NFHS-4 | % Diff | NFHS-3 | NFHS-4 | % Diff |  |
| **North India** | Delhi | 0.5 | 0.9 | 80.0 | 0.2 | 0.8 | 300.0 | 0.3 | 1 | 233.3 | 0.5 | 0.9 | 80.0 |  |
|  | Haryana | 1.4 | 2.3 | 64.3 | 1.5 | 2.7 | 80.0 | 1.1 | 2.1 | 90.9 | 1.4 | 2.3 | 64.3 |  |
|  | Punjab | 1.6 | 1.3 | -18.8 | 1.6 | 1.4 | -12.5 | 1.6 | 1.2 | -25.0 | 1.6 | 1.3 | -18.8 |  |
|  | Jammu & Kashmir | 0.2 | 0.4 | 100.0 | 0.3 | 0.6 | 100.0 | 0.4 | 1 | 150.0 | 0.3 | 0.6 | **100.0** |  |
|  | Uttarakhand | 0.6 | 0.3 | -50.0 | 0.4 | 0.3 | -25.0 | 0.4 | 0.3 | -25.0 | 0.6 | 0.3 | -50.0 |  |
|  | Himachal Pradesh | 0.1 | 0.2 | 100.0 | 0.1 | 0.3 | 200.0 | 0.1 | 0.3 | 200.0 | 0.1 | 0.2 | **100.0** |  |
| **Central India** | Uttar Pradesh | 17.8 | 14.8 | -16.9 | 13.6 | 14.6 | 7.4 | 15.4 | 12.4 | -19.5 | 17 | 14.4 | -15.3 |  |
|  | Rajasthan | 6.4 | 4.1 | -35.9 | 11.2 | 2.8 | -75.0 | 8.2 | 3.1 | -62.2 | 6.9 | 4 | -42.0 |  |
|  | Chhattisgarh | 1.8 | 2.5 | 38.9 | 1.5 | 1.9 | 26.7 | 1.7 | 2.3 | 35.3 | 1.7 | 2.3 | 35.3 |  |
|  | Madhya Pradesh | 7.9 | 6 | -24.1 | 6.8 | 6.6 | -2.9 | 8.8 | 5.1 | -42.0 | 7.8 | 5.9 | -24.4 |  |
| **West India** | Maharashtra | 8.1 | 6.1 | -24.7 | 1.8 | 2.9 | 61.1 | 10.4 | 5.7 | -45.2 | 7.8 | 6 | -23.1 |  |
|  | Gujarat | 3.7 | 4.5 | 21.6 | 3.8 | 4.4 | 15.8 | 6.1 | 5.9 | -3.3 | 4.4 | 4.8 | 9.1 |  |
|  | Goa | 0.1 | 0.1 | 0.0 | 0 | 0.1 | 0 | 0.1 | 0.1 | 0.0 | 0.1 | 0.1 | 0 |  |
| **South India** | Andhra Pradesh | 8.1 | 11.1 | 37.0 | 3 | 7.3 | 143.3 | 6.6 | 11.0 | 66.7 | 7.5 | 10.6 | 41.3 |  |
|  | Karnataka | 2.9 | 3 | 3.4 | 1.9 | 4.4 | 131.6 | 2.6 | 4.3 | 65.4 | 2.9 | 3.3 | 13.8 |  |
|  | Tamil Nadu | 7.2 | 12.2 | 69.4 | 1.8 | 10.7 | 494.4 | 6.2 | 14.5 | 133.9 | 6.7 | 12.5 | 86.6 |  |
|  | Kerala | 1.1 | 1.6 | 45.5 | 1.2 | 2.1 | 75.0 | 1.5 | 2.3 | 53.3 | 1.2 | 1.8 | 50.0 |  |
| **East India** | West Bengal | 8.3 | 8.3 | 0.0 | 19.7 | 9.3 | -52.8 | 7 | 7.9 | 12.9 | 9.4 | 8.3 | -11.7 |  |
|  | Orissa | 3.6 | 3.8 | 5.6 | 5.7 | 4.3 | -24.6 | 4.7 | 2.9 | -38.3 | 3.9 | 3.8 | -2.6 |  |
|  | Bihar | 12.2 | 11.1 | -9.0 | 15.4 | 16.5 | 7.1 | 10.2 | 11.9 | 16.7 | 11.8 | 10.9 | -7.6 |  |
|  | Jharkhand | 2.6 | 2.7 | 3.8 | 3.4 | 2.9 | -14.7 | 3 | 1.8 | -40.0 | 2.7 | 2.5 | -7.4 |  |
| **North**  **East India** | Assam | 2.9 | 1.8 | -37.9 | 3.9 | 1.6 | -59.0 | 2.5 | 1.9 | -24.0 | 2.9 | 1.9 | -34.5 |  |
|  | Tripura | 0.4 | 0.4 | 0.0 | 0.7 | 0.5 | -28.6 | 0.5 | 0.4 | -20.0 | 0.4 | 0.4 | 0.0 |  |
|  | Arunachal Pradesh | 0.1 | 0.1 | 0.0 | 0.1 | 0.1 | 0 | 0.1 | 0.1 | 0.0 | 0.1 | 0.1 | 0.0 |  |
|  | Manipur | 0.2 | 0.3 | 50.0 | 0.3 | 0.3 | 0 | 0.1 | 0.2 | 100.0 | 0.2 | 0.3 | 50.0 |  |
|  | Meghalaya | 0.1 | 0.1 | 0.0 | 0 | 0.1 | 0 | 0.1 | 0.1 | 0.0 | 0.1 | 0.2 | **100.0** |  |
|  | Mizoram | 0 | 0 | 0 | 0 | 0 | 0 | 0 | 0 | 0 | 0 | 0 | 0 |  |
|  | Nagaland | 0.1 | 0 | -100.0 | 0 | 0.1 | 0 | 0.1 | 0.1 | 0 | 0.1 | 0 | -100.0 |  |
|  | Sikkim | 0 | 0 | 0 | 0 | 0 | 0 | 0 | 0 | 0 | 0 | 0 | 0 |  |
